# Early reduced myocardial diastolic function in children and adolescents with type 1 diabetes mellitus a population-based study

**DOI:** 10.1186/s12872-016-0288-1

**Published:** 2016-05-25

**Authors:** Leif Brunvand, Drude Fugelseth, Knut Håkon Stensaeth, Knut Dahl-Jørgensen, Hanna Dis Margeirsdottir

**Affiliations:** Department of Pediatrics, Section for Heart Diseases, Oslo University Hospital, Oslo, Norway; Department of Neonatal Intensive Care, Oslo University Hospital, Ullevål, Oslo, Norway; Institute of Clinical Medicine, Faculty of Medicine, University of Oslo, Oslo, Norway; Department of Radiology and Nuclear medicine, Institute of Circulation and Imaging, Norwegian University of Science and Technology and St Olavs University Hospital, Trondheim, Norway; Department of Pediatrics, Akershus University Hospital, Nordbyhagen, Norway

**Keywords:** Diabetes type-1, Tissue Doppler imaging, Echocardiography, Diastolic myocardial function, Body mass index, Blood pressure

## Abstract

**Background:**

Reduced diastolic myocardial function is an early sign of diabetic cardiomyopathy. The aim of this study was to test the hypothesis that children and adolescents with type 1 diabetes mellitus (T1D), but without other known complications, have early reduced diastolic myocardial function diagnosed with echocardiographic color tissue Doppler imaging (cTDI).

**Methods:**

cTDI examination was carried out in 173 T1D patients and 62 age-matched controls. The T1D-patients were 8–18 years old with (mean (SD)) diabetes duration of 5.6 (3.4) years and HbA1c of 8.4 (1.3). All were treated with either insulin pumps or 4–6 daily insulin injections. cTDI early (E’) and late (A’) peak diastolic velocities and systolic peak velocity were measured from the lateral, septal, anterior and posterior mitral annulus and from the lateral tricuspidal annulus.

**Results:**

Myocardial diastolic function was reduced in the T1D-patients with higher peak A’-velocity and lower E’/A’-ratio in all registrations. Overall mean (SD) mitral E’/A’-ratio was 2.3 (0.5) in T1D and 2.7 (0.6) in the controls (*p* < 0001). The overall mitral E’/A’-ratio was negative associated with blood pressure (BP) and body mass index (BMI). Stratifying all participants into three groups according to BMI (<25, 25–75, >75 centile, respectively), the T1D had lower E’/A’-values in all stratified groups, except for in the highest BMI-group where both T1D and controls had the lowest E’/A’-ratio. Systolic function did not differ in any of the measurements. There were no associations with sex, diabetes duration, carotid artery intima-media-thickness, vessel elasticity or HbA1c.

**Conclusion:**

Diabetic children and adolescents using modern intensive insulin treatment had echocardiographic signs of reduced diastolic myocardial function despite short duration of disease. The reduced function was associated with higher BP and higher BMI.

## Background

Clinical and experimental studies have demonstrated evidence of diabetic cardiomyopathy independent of coronary artery disease and hypertension [1, 2]. Diabetes cardiomyopathy is strongly associated with adverse prognosis in adults [3–5]. Diabetic cardiomyopathy can affect diastolic function, systolic function, or both [1]. Reduced systolic and diastolic function has been demonstrated in adults with diabetes without known diabetic cardiomyopathy [6–8]. However, the process probably starts in early childhood and may affect both ventricles [9, 10]. The preclinical stage is suggested to primarly consist of reduced diastolic function. It may be demonstrated with tissue Doppler imaging (TDI) techniques long before it can be detected by 'conventional echocardiographic methods [11–14]. TDI seems to be the most powerfull echocardiographic method to evaluate diastolic function in T1D [15, 16]. Diabetic cardiomyopathy has also been assosciated with reduced elasticity of the great arterial vessels [17].

We hypothesized that diastolic myocardial dysfunction is present at an early stage of T1D despite treatment with modern intensified insulin regimens and no signs of microvascular complications. The aim of this study was to explore the diastolic myocardial function in children and adolescents with type 1 diabetes compared to healthy controls.

## Methods

### Study population

All children and adolescents with T1D aged 8–18 years in Health Region South-East in Norway (*n* = 800) were invited to participate in a study focusing on cardiovascular risk factors and early signs of atherosclerosis. Forty percent of the invited (*n* = 314) agreed to participate and were compared with 118 age-matched healthy control subjects. The diabetic cohort was found to be representative of the childhood T1D-population in Norway. None of the diabetic patients had signs of renal, retinal or neurological complications of the disease and all were using intensified insulin treatment (more than four daily insulin injections or insulin pumps). Results from the main study have been published earlier [18]. Out of this main study population a sub-study was conducted to evaluate the myocardial function with echocardiographic TDI examination. The enrolment in this sub-study was at random and depended only on the availability of at least one of the two pediatric cardiologists to perform the echocardiographic examination on the same day as the other investigations were taking place. All participants that were asked to participate in this echocardiographic sub-study agreed, and so 173 T1D and 62 control subjects were included.

### Ethical issues

Written informed consent was obtained from all the parents and from the participants when older than 12 years of age. The study protocols were approved by the Regional Committee for Medical and Health Research Ethics South East, Norway and the Norwegian Social Science Data Services.

### Baseline characteristics and laboratory measurements

The baseline measurements of blood pressure (BP), weight and height were recorded, and body mass index (BMI) was calculated as the ratio of the individual’s body weight to the square of the height. Arterial BP was measured according to the National High Blood Pressure Education Program Working Group on High Blood Pressure in Children and Adolescents [19].

Glycated hemoglobin (HbA1c) was determined for all participants by liquid chromatography (Variant; Bio-Rad, Richmond, CA) at the Department of Clinical Chemistry at Oslo University Hospital, Aker. The normal reference range was 4.1–6.4 (±2 %), and the intra-assay coefficient of variation was <3 %. Annual HbA1c values were available from the Norwegian Childhood Diabetes Registry since 2001 or from the time of diagnosis for the majority of the patients. Mean HbA1c was calculated from all the HbA1c values available for each patient [18].

### Echocardiography

Two experienced pediatric cardiologists (DF and LB) performed all echocardiographic examinations by use of a Vivid 7 scanner (GE, Horten, Norway) with a standard phase-array multi-frequency transducer (5 S probe, GE, Horten, Norway) transmitting at 2.4 MHz. The children were awake and relaxed in a left semi-lateral supine position. A structured sequential two-dimensional echocardiogram was made from subcostal, apical, parasternal long and short-axis and supra-costal views to rule out structural heart defects [20]. Tissue Doppler images (TDI) of the left ventricle (LV) and right ventricle (RV) were recorded from standard apical two and four chamber views using a tissue velocity range of ±0.22m/s. In all images, the sector angle was 30° or less and the tissue Doppler frame rate was 170–220/s. For each view, three to five consecutive cardiac cycles were recorded. ECG was recorded simultaneously. Septum length was measured in B-mode four-chamber view from the apical epicardium to the sepal attachment of the mitral valve.

From the apical 4-chamber view, the peak pulsed blood flow mitral valve annulus early diastolic (E) and late diastolic (A) velocities were measured with the sample volume positioned at the tip of the mitral leaflets and at an angle as parallel to mitral flow as possible.

EchoPac software (EchoPac PC SW, GE, Horten, Norway) was used for post processing of the TDI indices by color TDI (cTDI). cTDI early diastolic peak velocity (E’), atrial peak velocity (A’), E’/A’-ratio and systolic peak velocity were analyzed from the lateral, septal, anterior and posterior mitral annulus and from the lateral tricuspidal annulus. The region of interest (ROI) was set to 3x1 mm. Measurements were made with the ROI placed stationary in the ultrasound sector at the atrioventricular valve insertion. If artifacts occurred, the ROI was slightly repositioned in order to minimize background noise. If background noise could not be reduced, the recording was defined as poor quality and excluded from further analysis. Peak systolic velocity (cTDI S´) was measured at the maximum height of the systolic velocity curve.

#### *Common carotid artery* ultrasound

A standard ultrasonic protocol was used for measuring the common carotid artery intima-media thickness (cIMT) and the elasticity of the artery [21], as described earlier [18]. The distensibility reflects the mechanical load on the artery wall and is generally expressed as changes in lumen cross-sectional area (A) during the cardiac cycle. The distensibility coefficient (DC) as a measure of elasticity was calculated from the expression (DC = (ΔA/A)/ Δ*P* = ((d + Δd)2-d2)/d2/Δp). Δp represents the change in pulse pressure, d the end-diastolic diameter (including the intima-media complex) and Δd the change in diameter from diastole to systole, assuming a circular lumen cross-section of the vessel [18].

### Statistical analysis

Demographic and clinical data are presented as percentages or means with their standard deviations. Means were compared with student’s t-test when the data was normally distributed, otherwise with Mann–Whitney U test. The participants were stratified with respect to BMI into three different groups; group I (levels <25 centile), group II (levels between 25–75 centile) and group III (levels >75 centile). The differences within the stratified groups were tested with student’s t- test or Mann–Whitney U test. Correlation was tested with Pearson’s linear regression method.

## Results

The demographic data and baseline measurements of HbA1c, systolic and diastolic BP, cIMT as well as DC calculations are presented in Table 1. The echocardiographic cTDI and blood flow velocity parameters are presented in Tables 2 and 3. The calculated E´/A´-ratio and BP stratified according to the three BMI-groups are presented in Table 4. Echocardiographic recording defined as poor quality was excluded from further analysis. Complete datasets were achieved in 146 of the 173 T1D-participant and in 56 of the 62 controls.

**Table 1:** Demographic and metabolic parameter presented as % or mean (SD)

Parameter	T1D	control	*p* value
	*n* = 173	*n* = 62	
Age (years)	13.6 (2.8)	13.0 (2.6)	ns
Sex (boy/girl)	86/87	21/41	
Height (cm)	159.8 (14.6)	154.4 (14.1)	0.01
Weight (kg)	54.8 (17.0)	45.8 (14.4)	<0.001
Body mass index (kg/m^2^)	20.9 (4.1)	18.8 (3.6)	<0.001
Systolic blood pressure (mmHg)	101.1 (9.7)	96.5 (10.5)	0.002
Diastolic blood pressure (mmHg)	61.3 (8.4)	56.8 (7.4)	<0.001
Duration of T1D (years)	5.6 (3.4)		-
Serum creatinine (μmol/l)	53.0 (10.5)	53.1 (10.3)	ns
Urinary albumin/creatinine	1.2 (2.1)	1.5 (2.3)	ns
HbA1c (%)	8.4 (1.3)	5.3 (0.29)	
Mean HbA1c (%)	8.0 (0.96)		
CRP(mg/L)	1.9 (3.7)	1.0 (3.1)	ns
cIMT (mm)	0.48 (0.05)	0.48 (0.05)	ns
DC (kPa^−1^)	0.044 (0.02)	0.046 (0.01)	ns

**Table 2:** Measurement of cardiac blood flow and cTDI

	T1D	control	*p* value
	*n* = 146	*n* = 56	
MV E (m/s)	0.93 (0.16)	0.92 (0.16)	ns
MV A (m/s)	0.43 (0.11)	0.41 (0.11)	ns
E/A	2.3 (0.7)	2.4 (0.9)	ns
E/E’	8.1 (1.6)	7.6 (1.4)	ns
Septum length (cm)	8.7 (1.1)	8.2 (1.1)	0.007
MV lateral E’/A’	3.2 (1.3)	3.6 (1.4)	0.02
MV septum E’/A’	2.1 (0.7)	2.3 (0.6)	0.007
MV inferior E’/A’	2.3 (0.6)	2.7 (0.9)	<0.001
MV anterior E’/A’	2.3 (0.7)	2.8 (0.6)	<0.001
TV E’/A’	1.6 (0.6)	1.8 (0.6)	0.009

Mitral valve (MV), tricuspidal valve (TV), maximal early diastolic flow across the valve (E), maximal late diastolic flow across the valve (A), maximal early diastolic velocity of the annulus (E’), maximal late diastolic velocity of the annulus (A’), maximal systolic velocity of the annulus (S’). All the measurements were performed with the probe in a standard apical position. All data presented in mean (SD)

**Table 3:** Maximal velocities

	T1D	control	*p* value
	*n* = 146	*n* = 56	
MV S’ (cm/s)	7.0 (1.0)	6.9 (1.0)	ns
MV E’ (cm/s)	11.7 (1.4)	12.1 (1.5)	ns
MV A’ (cm/s)	−5.2 (1.0)	−4.6 (1.0)	0.006
MV E'/A’	2.3 (0.5)	2.7 (0.6)	<0.001
TV S’ (cm/s)	10.6 (1.6)	10.8 (1.7)	ns
TV A’ (cm/s)	−8.1 (2.5)	−7.2 (2.3)	0.03
TV E’ (cm/s)	−11.7 (2.1)	−12.0 (1.8)	ns

Mitral valve (MV), tricuspidal valve (TV), maximal early diastolic flow across the valve (E), maximal late diastolic flow across the valve (A), maximal early diastolic velocity of the annulus (E’), maximal late diastolic velocity of the annulus (A’), maximal systolic velocity of the annulus (S’). All the measurements were performed with the probe in a standard apical position. All data presented in mean (SD)

**Table 4:** Mean MV E’/A’-ratio, mean TV E’/A’ ratio and mean blood pressure (BP) after stratifying the participants according to body mass index (BMI). Data presented as mean values

	T1D	control	*p* value
Group I (BMI 14–18 kg/m^2^)	*n* = 33	*n* = 18	
MV E’/A’	2.4	2.9	0.005
TV E’/A’	1.7	2.0	
BP (mmHg)	95/58	93/56	ns
Group II (BMI 19–22 kg/m^2^)	n = 69	n = 31	
MV E’/A’	2.4	2.8	0.007
TV E’/A’	1.6	1.8	
BP (mmHg)	98/59	97/55	ns
Group III (BMI 23–35 kg/m^2^)	n = 44	n = 7	
MV E’/A’	2.1	2.1	ns
TV E’/A’	1.5	1.2	ns
BP (mmHg)	110/67	104/65	ns

Myocardial diastolic function was reduced in the T1D. The mean value for E’/A’-ratio was lower in T1D population compared to the controls in all registrations from the mitral and the lateral tricuspidal annulus ring (Tables 2 and 3). The overall E’/A’-ratio from the mitral annulus ring was 2.3 in the diabetic group and 2.7 in the controls (*p* < 0001) (Table 3). The mitral E’/A’-ratio was also negative associated with systolic and diastolic BP and BMI, but there were no associations with sex, years after the diagnosis of diabetes and HbA1c. There were no differences in the mean mitral value for E/E’-ratio or in systolic function (S´) between the diabetic and non-diabetic group.

The T1D population stratified into BMI group I and II had significant lower E’/A’-ratio than the controls. Those in the highest stratified group also had the lowest E’/A’-ratio, but with no differences between the diabetic and non-diabetic population. There were no differences between diabetic and non-diabetic groups regarding systolic or diastolic BP, septum length or height. There were no differences regarding sex, age, diabetes duration and HbA1c, nor any differences regarding systolic function (S´).

There were no association between E’/A’-ratio and cIMT and no differences between the diabetic and non-diabetic population regarding cIMT. However, a small but significant positive correlation was found between DC and E’/A’-ratio for the whole group (r^2^ = 0.05, *p* = 0.003). Separated into those with diabetes or not, there was still an association in the T1D group (r^2^ = 0.06, *p* = 0.003), but not in the control group.

## Discussion

The main finding in this study was that children and adolescents with T1D had lower E’/A’-ratio in all measurements from the mitral and lateral tricuspidal annulus ring. This confirms that even young children and adolescence with insulin dependent diabetes, but with no signs of diabetic comorbidity, have a small, but significant reduction of the diastolic myocardial function [22, 23]. In contrast to one earlier study [24], we could not find any differences between sexes. This is in accordance with a study of long-term T1D patients from Oslo [25], and also with other studies in children and adolescents [26].

The mean mitral E’/A’-ratio was negatively associated with BMI. This difference was still present in the lower and in middle BMI groups when stratified into different groups of BMI. Both the T1D and controls in the highest BMI group (group III) had the lowest E’/A’-ratio of all, but there was no differences between the control and diabetic group (Table 4). However, the result is uncertain due to lack of power in this control group (Table 4). A high BMI seems to be an independent risk factor for reduced diastolic function as also demonstrated by other authors [24, 26]. The small, but significant positive correlation between DC and E’/A’-ratio shows that E’/A’-ratio to some extent may reflect a reduced elasticity in both the atrial wall and the wall of the common carotid artery.

We did not find any associations between E’/A’-ratio and diabetes duration or HbA1c as demonstrated by others [6]. This may reflect the young age of the participants and the short duration of the disease in our study and that their diabetes seems to be under fair blood glucose control.

Interestingly we did not find any differences in E/E’-ratio. E/E’-ratio is a marker of elevated diastolic left ventricle and left atrium pressure [16]. This might reflect that the T1D-children in our study only had a small reduction in their diastolic function.

There were no differences in measured S’ between children with and without diabetes. S’ reflects the systolic longitudinal function of the heart and our findings is in harmony with other papers. It support the hypothesis that diabetes cardiomyopathy debuts with a reduced diastolic function, while the systolic function is preserved until a more advanced stage of the disease.

## Conclusion

Children and adolescents with type 1 diabetes using modern intensive insulin treatment had echocardiographic signs of reduced diastolic myocardial function despite short duration of disease. The reduced function was associated with higher blood pressure and higher body mass index both in the diabetic and in the non-diabetic groups.

## Abbreviations

A, peak late mitral valve diastolic blood flow velocity; A’, peak late diastolic annulus tissue Doppler velocity; BMI, body mass index; BP, blood pressure; cIMT, carotid intima-media thickness; cTDI, color tissue Doppler imaging; DC, distensibility coefficient; E, peak early mitral valve diastolic blood flow velocity; E’, peak early diastolic annulus tissue Doppler velocity; MV, mitral valve; ROI, region of interest; S’, peak systolic annulus tissue Doppler velocity; T1D, type 1 diabetes mellitus; TDI, tissue Doppler imaging; TV, tricuspidal valve.
